# Tumor Necrosis Factor Receptor-Associated Periodic Syndrome (TRAPS) with a New Pathogenic Variant in *TNFRSF1A* Gene in a Family of the Adult Male with Renal AA Amyloidosis—Diagnostic and Therapeutic Challenge for Clinicians

**DOI:** 10.3390/jcm10030465

**Published:** 2021-01-26

**Authors:** Jolanta Zegarska, Ewa Wiesik-Szewczyk, Ewa Hryniewiecka, Beata Wolska-Kusnierz, Dariusz Soldacki, Magdalena Kacprzak, Agnieszka Sobczynska-Tomaszewska, Kamila Czerska, Pawel Siedlecki, Karina Jahnz-Rozyk, Ewa Bernatowska, Radoslaw Zagozdzon, Leszek Paczek

**Affiliations:** 1 Department of Immunology, Transplant Medicine and Internal Diseases, Medical University of Warsaw, 59 Nowogrodzka St., 02-006 Warsaw, Poland; jzegarska@wum.edu.pl (J.Z.); elhryniewiecka@gmail.com (E.H.); 2 Department of Internal Medicine, Pulmonology, Allergy and Clinical Immunology, Central Clinical Hospital of the Ministry of National Defense, Military Institute of Medicine in Warsaw, 128 Szaserów St., 04-141 Warsaw, Poland; ewa.w.szewczyk@gmail.com (E.W.-S.); dariusz.soldacki@gmail.com (D.S.); kjrozyk@wim.mil.pl (K.J.-R.); 3 Department of Immunology, Children’s Memorial Health Institute, 20 Dzieci Polskich Ave., 04-730 Warsaw, Poland; bwolska@interia.pl (B.W.-K.); ewa.bernatowska@gmail.com (E.B.); 4 Department of Clinical Immunology, Medical University of Warsaw, 59 Nowogrodzka St., 02-006 Warsaw, Poland; 5 MEDGEN Medical Centre, 9a Wiktorii Wiedenskiej St., 02-954 Warsaw, Poland; mk@medgen.pl (M.K.); agnieszka.sobczynska@medgen.pl (A.S.-T.); kamila.czerska@medgen.pl (K.C.); 6 Department of Bioinformatics, Institute of Biochemistry and Biophysics, Polish Academy of Sciences, 5a Adolfa Pawinskiego St., 02-106 Warsaw, Poland; psiedlecki@gmail.com; 7 Department of Systems Biology, University of Warsaw, 1 Miecznikowa 1., 02-096 Warsaw, Poland

**Keywords:** new genetic variant, monogenic autoinflammatory syndrome, diagnostic delay, anakinra, damage index, genetic inheritance, personalized therapy

## Abstract

Tumor necrosis factor receptor-associated periodic syndrome (TRAPS) belongs to systemic autoinflammatory diseases (AIDs). Many of these syndromes are genetically conditioned and can be inherited. Diagnosis relies on clinical symptoms and should be confirmed by genetic testing. One of the most serious complications is AA amyloidosis. We present the diagnostic route of a 33-year-old male with AA amyloidosis and his children, leading to diagnosis of monogenic autoinflammatory syndrome, confirmed by genetic analysis. A novel variant of the in-frame insertion type in one allele of *TNFRSF1A* gene was found by whole exome sequencing and confirmed by Sanger sequencing, which allowed a diagnosis of TRAPS. Three-dimensional modeling was used to assess the structural changes introduced into TNFR1 molecule by the insertion. The analysis of the 3D model revealed that accommodation of the 4AA insert induces misalignment of three cysteine bridges (especially the C70-C96 bridge) in the extracellular domain, leading to putatively misfolded and improperly functioning TNFR1. Three of the patient’s daughters inherited the same variant of the *TNFRSF1A* gene and presented TRAPS symptoms. TRAPS is a very rare disease, but in the presence of suggestive symptoms the genetic diagnostic workout should be undertaken. Early diagnosis followed by appropriate clinical management can prevent irreversible complications.

## 1. Introduction

Monogenic autoinflammatory diseases (AIDs) cover a spectrum of syndromes, which lead to chronic or recurrent inflammation caused by activation of the innate immune system, typically in the absence of high autoantibody titers [1]. The four most common monogenic AIDs are: NLRP3-associated autoinflammatory disease (NLRP3-AID), familial Mediterranean fever (FMF), mevalonate kinase deficiency (MKD), and tumor necrosis factor receptor-associated periodic fever syndrome (TRAPS).

TRAPS is an autosomal dominant disease, caused by mutations in *TNFRSF1A* gene which encodes the protein named tumor necrosis factor receptor 1 (TNFR1), which plays a crucial role in the inflammation and apoptosis [1]. The estimated prevalence of TRAPS is one per million. Symptoms include recurrent episodes of fever, lasting about 3 weeks, abdominal, chest, and muscle pains, skin rash, typically found on the limbs, periorbital edema, and joint pain [2]. Inflammatory markers are always elevated during acute episodes. The onset of the disease may occur at any age, from infancy to late adulthood, but most patients have their first episode in childhood. In most cases, relatives are affected, and positive family history strongly supports the diagnosis. AA amyloidosis is the main long-term complication of TRAPS. Depending on the genetic variant, it is estimated that 2–24% of untreated patients with TRAPS would develop AA amyloidosis [2,3,4,5], usually in mid-adulthood, but the risk of this complication has significantly been decreased by modern anti-inflammatory therapies [6]. The evaluation of patient with systemic AA amyloidosis, in whom an obvious cause cannot be identified, is a challenge in clinical practice, as there are over 100 diseases associated with AA amyloidosis. Among them, strong association is reported for AIDs. The correlation is not unexpected, because autoinflammatory syndromes often cause the long-standing inflammation [7]. However, due to low awareness and heterogenous presentation, especially in sporadic cases, the diagnosis of AIDs might be overlooked. Indeed, long diagnostic delay is usually reported. Importantly, the proper diagnosis allows for avoiding the complications and progressive, irreversible organ damage. Moreover, worse prognosis and unfavorable outcomes of renal transplantation is reported in AA amyloidosis in the course of AIDs [8].

The therapies include corticosteroid therapy, non-steroidal anti-inflammatory drugs (NSAIDs) during flares or a treatment based on biological agents, depending on the requirements of the particular patient and disease severity [9,10]. Targeted anti-inflammatory treatment is the only option in preventing further systemic deposition of amyloid and recurrence of amyloidosis in transplanted organ.

The aim of the study was the identification of molecular basis and inheritance pattern of disease in an adult male with AA amyloidosis and episodes of fever suspected of AID, as well as in the members of his family.

## 2. Materials and Methods

### 2.1. Patients

4 patients were included in the study—proband and all his 3 children (daughters). At the admission, the proband was 33-year-old Caucasian male, who was referred for nephrology consultation because of proteinuria, chronic renal failure with estimated glomerular filtration rate (eGFR) 69 mL/min./1.73 m^2^, and biopsy-proven kidney AA amyloidosis.

### 2.2. Methods of Molecular Analysis

Next-generation sequencing (NGS). Whole exome sequencing (WES) was performed. Patient’s genomic DNA was extracted from the whole blood sample, and sequencing library was prepared according to Agilent Sure-Select Human All Exon V5 protocol. The enriched DNA libraries were sequenced by the Illumina HiSeq4000 instrument (Illumina, Inc., Sand Diego, CA, USA). All procedures for exome sequencing were conducted by Macrogen (Seoul, Korea). Raw sequencing reads were mapped to the reference genome using BWA [11]. Duplicates were removed using Picard software (Broad Institute, Cambridge, MA, USA), and variants were named using Samtools software (SourceForge, San Diego, CA, USA). Variants in 289 genes connected with autoinflammatory diseases were analyzed (the list of genes [12] is presented in Supplementary Table S1). The following in silico prediction software programs were used to assist with interpretation of pathogenicity of detected variant: Alamut visual v 2.9.0 (Interactive biosoftware, SOPHiAGENETICS, CH-1025 Saint Sulpice, Switzerland). The presence of the variant in control populations was checked in 1000Genomes [13], the Exome Variant Server [14], and the Exome Aggregation Consortium (Broad Institute) and gnomAD (Broad Institute).

### 2.3. 3D Protein Modeling

The newly identified AHRH insertion was manually introduced into the structure of TNFR1 (Tumor necrosis factor receptor superfamily member 1A; PDB:1FT4) with the MAV (Multalign Viewer) module of UCSF (University of California San Francisco, CA, USA) Chimera software (v 1.14) [15]. Next, the Modeler software [16] was used to generate 5 different models of the modified structure. The resulting models were validated with zDOPE (Discrete Optimized Protein Energy score [17]). Final model was subjected to a short minimization procedure (MMTK—Molecular Modelling Toolkit, Center for Molecular Biophysics, CNRS-Orleans, France) with Amber ff14SB force field, 100 steps of steepest descent and 10 steps conjugate gradient) to lessen the sterical constraints introduced by the modeling procedure.

## 3. Results

### 3.1. Patients

#### 3.1.1. Patient 1—Proband

33-year-old Caucasian male was referred for nephrology consultation because of proteinuria 3.2–5.7 g/day; chronic renal failure with estimated glomerular filtration rate (eGFR) 69 mL/min./1.73 m^2^ and biopsy-proven kidney AA amyloidosis. On admission, he complained of chronic fatigue; otherwise, he denied any other symptoms. His family medical history was reported as irrelevant. His comorbidities were as follows: arterial hypertension, hypercholesterolemia, normocytic anemia. There were no clinically relevant findings during physical examination, except for the post-appendicitis scar in right lower quadrant of the abdomen.

In routine laboratory tests, the erythrocyte sedimentation rate (ESR) was 47 mm/h (reference range: 0–12 mm/h), and serum concentration of C-reactive protein (CRP) 61.17 mg/L (reference range: up to 9 mg/L), serum amyloid A (SAA) 60.9 mg/L (reference range: up to 6.4 mg/L). Laboratory work-up for anti-nuclear (ANA), anti-neutrophil cytoplasmic (ANCA) antibodies and rheumatoid factor were negative. Blood and urine cultures were also negative.

Detailed analysis of his past medical history revealed that from the age of 7 the patient reported recurrent episodes of abdominal pain with fever up to 40 °C, upper respiratory tract symptoms (pharyngitis, tonsillitis), arthralgia of elbow, knee, and wrists and elevated inflammatory parameters. These episodes were recurring irregularly, at least twice a year, with fever lasting 7–10 days and abdominal pain lasting 10–14 days. He was repeatedly hospitalized and suspected of infections (at the age of 7), chronic endocarditis (aged 14), lambliasis with cholangitis (aged 13), or connective tissue diseases. Episodes of abdominal pain were the rationale for appendectomy (age 14). Inflammatory bowel disease and chronic pancreatitis were excluded. Despite extensive research, the reason for recurrent fevers was not identified. During adolescence, recurrent acute episodes stopped, but chronic low-grade fever and fatigue persisted. At the age of 28, the patient was diagnosed with proteinuria, kidney function impairment, and arterial hypertension. Kidney biopsy specimen revealed AA amyloidosis.

Based on the medical history, AIDs were included as a potential cause for AA amyloidosis. Initial suspicion of FMF was made and treatment with colchicine 0.5 mg/day twice a day was ordered, with short-time improvement of ESR and CRP. An attempt to increase colchicine dosage to 0.5 mg three times a day was unsuccessful due to diarrhea. Moreover, genetic tests for FMF did not reveal any known pathogenic variants in exons 2, 3, and 10 of *MEFV* gene. Due to unsatisfactory effectiveness of the treatment and sustained suspicion of AID as the cause of AA amyloidosis, the genetic diagnostic work-up was continued by next-generation sequencing (NGS).

#### 3.1.2. Remaining Patients

##### Patient 2

At the age of 7, Patient 1’s daughter had been referred for consultation due to episodes of fever up to 39 °C lasting for 6–10 days accompanied by severe abdominal pain, lymphadenopathy (cervical, inguinal) and recurrent pharyngitis. Flares have been occurring irregularly 4–5 times a year since the age of 2. Her physical examination between episodes was normal. She had constantly elevated SAA during and between febrile flares (67–813 mg/dL). Until the diagnosis of the father, her symptoms were treated as typical childhood infections.

##### Patient 3

At the age of 9, the second daughter had presented fever episodes. Her symptoms have been sporadic and mild, and lasted approx. 10 days. She responded well to antipyretic treatment. Her acute inflammatory reactants were high during flares (CRP up to 6.0 mg/dL), and between episodes were within the reference range (SAA 3.7 mg/dL).

##### Patient 4

At the age of 17, the oldest daughter presented with severe abdominal pain, low-grade fever 37.5 °C. No abnormalities were found on additional investigation except for abnormal inflammatory markers (CRP 8.2 mg/dL). Symptoms persisted for 3 weeks, without response on NSAID and resolved spontaneously. Otherwise, her medical history was unremarkable. Her acute inflammatory reactants between episodes were within the reference range (SAA 0.8 mg/dL, CRP 0.2 mg/dL).

### 3.2. Genetic Results

#### Genetic Variant Identification

**Figure 1:**
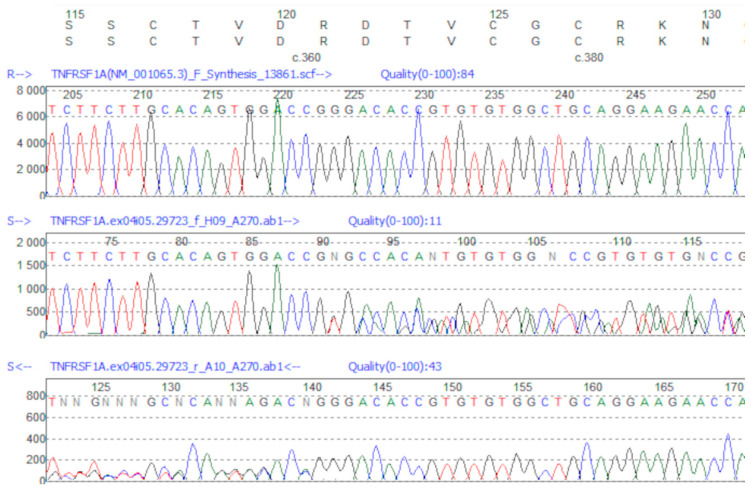
Sanger sequencing of NM_001065.5:c.362_363insTGCAAGACACAG variant. The alignment to wild sample sequence is visualized using Mutation Surveyor Software, v.4.0.7 (Softgenetics, State College, PA 16803, USA). The upper panel represents the reference sequence, underneath—Sanger sequencing of mutated sample—from forward (middle panel) and from reverse (bottom panel) primers.

As the result of WES analysis of Patient 1, the novel variant of the insertion type: c.362_363insTGCAAGACACAG in one allele of *TNFRSF1A* gene was identified (Supplementary Figure S1). Then, the NM_001065.5:c.362_363insTGCAAGACACAG/p.(Arg121_Asp122insAlaArgHisArg) variant was confirmed by Sanger sequencing (Figure 1).

**Figure 2:**
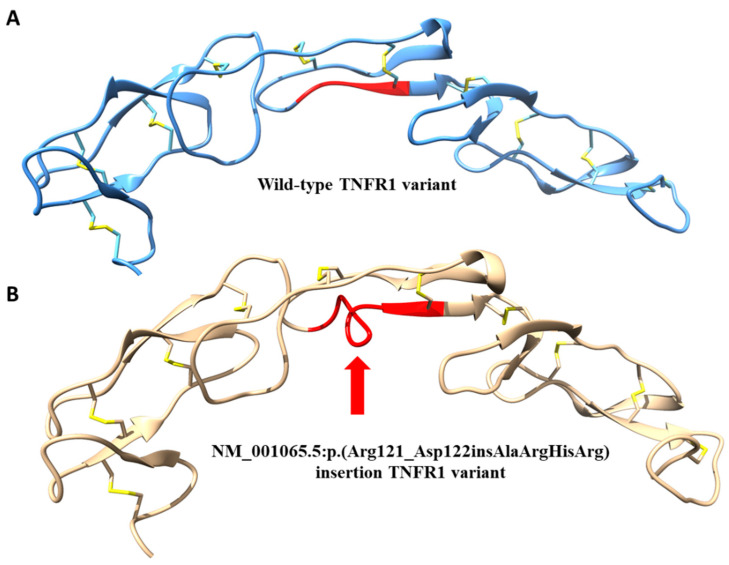
(**A**,**B**) Comparison between the structure of wild-type variant of TNFR1 protein (**A**) and modeling of the predicted misfolding (red arrow) resulting from the NM_001065.5: p.(Arg121_Asp122insAlaArgHisArg) insertion in the pathogenic variant of TNFR1 (**B**).

Because the NM_001065.5:p.(Arg121_Asp122insAlaArgHisArg) insert has been identified in the TNFR1 protein for the first time, we decided to assess the potential effects of this insertion on the protein structure by 3D modeling (Figure 2). The acquired 3D model suggested that the insert may influence three cysteine bridges, with the C70–C96 being the crucial one for correctly orienting CRD2 and CRD3 domains. Accommodation of the 4AA insert leads to misalignment cysteine bridges leading to putatively misfolded and improperly functioning TNFR1. Therefore, the NM_001065.5:p.(Arg121_Asp122insAlaArgHisArg) insert should be considered pathogenic. This notion warrants further investigations on the potential phenotypic changes induced by this novel TNFR1 variant under experimental conditions, preferably by comparison with some previously identified pathogenic variants, as exemplified in [18,19].

### 3.3. Management

From 19 December 2017, when biologic treatment for AIDs got reimbursement in Poland, Patient 1 started treatment with an interleukin 1 inhibitor—anakinra, 100 mg subcutaneously/day, as home therapy. At the beginning, he was taking the medication irregularly due to moderate local side effects (rush, skin burning). Patient 1 was re-educated about the aim of treatment, mode of application, and then continued the treatment regularly. After 3 months of follow-up, local side effects completely disappeared. In parallel, low-grade fever and fatigue resolved. Inflammatory parameters normalized: CRP to 0.1 mg/dL (reference range: 0.8 mg/dL), SAA to 0.6 mg/dL (reference range: to 0.64 mg/dL) and stay within reference range after 24 months treatment. His kidney function and proteinuria are stable (creatinine level 1.7 mg/dL, proteinuria 2.0 g/day).

From the patient’s perspective: during the 2-year follow-up despite initial doubts related to local reactions after administration of the preparation, we observed a very good compliance with the treatment principles. The applied targeted therapy not only resulted in the normalization of inflammatory parameters, but also eliminated the troublesome symptoms of excessive fatigue and the patient returned to his work on the farm. Currently, he feels free from burden of symptoms and from his point of view can give financial and personal support for his family members.

Patient 2 has started the treatment with IL-1 blocker (anakinra). Rapid response was observed with remission of symptoms within 2 days and normalization of lab results after 7 days (CRP 0.8 mg/dL; SAA 7.3 mg/dL). There were no flares during next 18 months of follow-up.

Patient 3 and Patient 4 remain asymptomatic and have currently normal laboratory results. They do not need chronic pharmacological treatment at present and are under thorough medical control.

## 4. Discussion

We present results of diagnostics that included NGS sequencing in a patient with kidney AA amyloidosis and all his children. The clinical presentation of the adult male patient was suggestive for AIDs, but not specific for a particular type of AID, including TRAPS, according to the clinical criteria acknowledged at the time of patient’s admission [20]. Predominant clinical symptoms were episodes of fever and severe abdominal pain in childhood, arthralgia and fatigue in adulthood, and AA amyloidosis as a long-term complication. Patient’s family history was negative, as well as the medical history in respect of migratory rash and periorbital edema—the more specific indicators for TRAPS [21]. According to the literature, the experts asked to indicate signs and symptoms regarded the most helpful in their practice for the diagnostics of TRAPS cited the following: recurrent long-lasting fever episodes, positive family history, periorbital edema, abdominal pain, myalgia, cutaneous rash, arthralgia, and monocytic fasciitis [22]. The onset of the disease in the reported male patient, as in most cases of TRAPS, was in his childhood, but at the time of diagnosis he presented the pattern typical for a chronic disease without inflammatory, acute flares. Notably, in his case it took approx. 29 years from the onset of the disease till the confirmation of diagnosis. Moreover, the proband developed AA amyloidosis and kidney failure, but it occurred quite early, in his twenties, while Lachman et al. found that the median age of developing of amyloidosis in TRAPS population was 43 years [5].

In clinical practice, there is a challenge to appropriately qualify the sporadic cases of AA amyloidosis for further genetic evaluation. Generally, when the clinical symptoms presented by the given patient are adherent with the diagnostic criteria for a specific AID (e.g., TRAPS), the genetic method of choice should be a targeted sequencing of the respective gene by the Sanger method. In the adult patient with AA amyloidosis reported hereby, an initial attempt was made of a targeted sequencing of selected exons of the *MEFV* gene, which proved unsuccessful. In the last decade, due to application of the next-generation sequencing (NGS), the genetic diagnosis in patients with AIDs has greatly improved and remarkable progress has been made in the genetic characterization of the undiagnosed patients and the sporadic cases [23]. Importantly, TRAPS is genetically heterogeneous. More than 140 *TNFRSF1A* variants have been recognized up to date [24]. *TNFRSF1A* gene comprises 10 exons. Most of the variants are single-nucleotide substitutions (95%), but deletions and insertions have also been reported. Most sequence variants lie within exons 2 to 4 and result in amino acid substitutions which disrupt important cysteine-cysteine disulfide bonds within the extracellular domain (cysteine-rich domains, CRDs). A single splicing mutation also perturbs the first CRD due to the insertion of 4 amino acids. The most common are low-penetrance variants R92Q and P46L. Another common variant is T50M. Generally, mutations that result in cysteine substitutions lead to higher penetrance of the clinical phenotype (93% versus 82% for non-cysteine residue substitutions), and increase the probability of developing amyloidosis (24% versus 2% for no cysteine residue substitutions) [2].

Hereby, to establish the diagnosis, we performed WES with subsequent analysis of genes connected with autoinflammatory diseases. As a result, we identified a novel *TNFRSF1A* variant of in-frame insertion type that putatively influences three cysteine bridges and the structure of TNFR1 protein leading to its dysfunction or loss of function. The variant was confirmed by Sanger analysis. To the best of our knowledge, this variant has not been reported up to date. Thus, the important question is whether this novel genetic variant is pathogenic. Notably, it was found in one allele, which corresponds to the fact that TRAPS is an autosomal dominant disease. Clinical arguments for its pathogenicity are that the same genetic variant was found in three symptomatic relatives (i.e., daughters). However, clinical spectrum varies among family members and leads to personalized management. Therefore, further research laboratory tests, e.g., in the cell culture models, are necessary to respond the question of pathogenicity with acceptable precision.

Management of TRAPS should be adjusted case by case. It can involve corticosteroid therapy, NSAIDs during flares or a treatment based on biological agents, mainly TNF receptor-IgG1 Fc fusion protein etanercept [25] or interleukin 1 inhibitors [9]. Current data indicate that anti-IL-1 compounds are the most effective drugs in patients with pathogenic variants [26]. Such treatment eliminates the clinical symptoms and inhibits progression of AA amyloidosis. Indeed, the efficiency of anti-IL-1 agents in TRAPS treatment has been proved. Anakinra, a recombinant and slightly modified version of the human interleukin 1 receptor antagonist protein or canakinumab, a human monoclonal antibody targeted at interleukin-1 beta can be used. In 47 TRAPS patients from the US, the European Union, and the eastern Mediterranean, treatment with anakinra versus anti-TNF agents as the first biologic therapy led to significantly higher clinical and biochemical responses [27]. In one adult with amyloidosis-related renal failure, anakinra led to the disappearance of manifestations of TRAPS and decrease of laboratory abnormalities, including proteinuria [28]. Moreover, this treatment is considered safe, as in the presented case the side effects were local and self-limiting. Canakinumab was effective in controlling and preventing fever flares in patients with TRAPS, colchicine-resistant FMF, and MKD [29,30]. In contrast to anakinra, which is administered daily, canakinumab can be given every 4–8 weeks. The limitation of its use is the high cost, and that at present time it is not reimbursed in Poland.

As mentioned above, management of TRAPS and qualification for biologic treatment should be personalized. In this study, three additional family members of the patient are symptomatic and have undergone diagnostic workout, but based on their symptoms and the estimated risk of end-organ damage, the goals and manners of management differ. The father (proband) is treated continuously with anakinra to stop the AA amyloidosis progression. His 7-year-old daughter received anakinra treatment before any damage accrual occurred, and in future it may be possible to pause the medication. Finally, two daughters are covered by watch-and-wait strategy, as flares were sporadic, and inflammatory markers are within reference range between attacks.

Our study has some limitations. We did not perform the functional studies on the newly identified variant. Moreover, the pathogenicity of the variant could be further supported by the assessment (e.g., by flow cytometry) of TNFR1 presence on the respective cells, and the serum concentrations of soluble TNFR1, TNF-α, and perhaps other proinflammatory cytokines. However, none these tests are routinely performed in the medical centers involved in the current study. Furthermore, given the information collected in this report, it would be preliminary to speculate what would be the pathophysiological and/or molecular mechanism(s) responsible for the pathogenicity of the newly identified variant. In theory, such effects may rely on the exaggerated activation of NF-κB pathway [18] and/or hyperresponsiveness to the proinflammatory stimuli [19], and then induction of subsequent inflammatory cascading. As mentioned, this should be further investigated in properly designed functional studies. Despite its limitations, our study supports the notion that in cases of AA amyloidosis of unknown origin, the monogenic AID, including TRAPS, should be considered.

## 5. Conclusions

TRAPS is a very rare disease and can be overlooked in differential diagnosis, especially if clinical picture is not specific. The diagnosis is usually delayed leading to organ damage with the most serious complications, i.e., the development of secondary inflammatory amyloidosis and kidney failure. In diagnosis, the crucial role is played by the genetic testing, preferably by NGS. Hereby, in an adult male with AA amyloidosis and his family members we identified a novel in-frame insertion-type pathogenic variant in one allele of *TNFRSF1A* gene that caused TRAPS. Setting up the correct diagnosis of TRAPS allows for the choice of a suitable treatment. Indeed, anti-IL-1 agents provide efficient and safe therapy that eliminates clinical symptoms and prevents the patients from the organ damage, which is exemplified in the current report.

## Data Availability

The novel molecular variant was 184 submitted to the ClinVar database (http://www.ncbi.nlm.nih.gov/clinvar; Submission ID: SUB6616942).

## Supplementary Materials

The following are available online at https://www.mdpi.com/2077-0383/10/3/465/s1, Figure S1: Next-generation (whole exome) sequencing and alignment visualization using Integrative Genomics Viewer (IGV), Table S1: Genes connected with inborn errors of immune system analyzed by NGS in 33-years-old man with AA renal amyloidosis.
