# Supplementary material for: Tumor Necrosis Factor Receptor-Associated Periodic Syndrome (TRAPS) with a New Pathogenic Variant in TNFRSF1A Gene in a Family of the Adult Male with Renal AA Amyloidosis—Diagnostic and Therapeutic Challenge for Clinicians
Source: J Clin Med. 2021 Jan 26;10(3):465. doi: 10.3390/jcm10030465 (PMC7865531; doi:10.3390/jcm10030465)

Supplementary Table S1. Genes connected with inborn errors of immune system analyzed by NGS in 33-years-old man with AA renal amyloidosis, please refer to the main text for details.

**Genes**
ACP5, ACTB, ADA, ADAM17, ADAR, AICDA, AIRE, AK2, AP1S3, AP3B2, APOL1, AR, ATM, B2M, BCL10, BLM, BLNK, BLOC1S6, BTK, C1QA, C1QB, C1QC, C1R, C1S, C2, C3, C4A, C4B, C5, C6, C7, C8A, C8B, C8G, C9, CARD11, CARD14, CARD8, CARD9, CASP10, CASP8, CCBE1, CD14, CD19, CD247, CD3D, CD3E, CD3G, CD40, CD40LG, CD46, CD59, CD79A, CD79B, CD81, CD8A, CEBPE, CECR1, CFB, CFD, CFH, CFHR1, CFHR2, CFHR3, CFHR4, CFHR5, CFI, CFP, CHD7, CIITA, CLPB, COLEC11, COPA, CORO1A, CR2, CSF2RA, CSF3R, CTLA4, CTSC, CXCR4, CYBA, CYBB, DCLRE1B, DCLRE1C, DKC1, DNASE1, DNASE1L3, DNMT3B, DOCK2, DOCK8, ELANE, EPG5, ERAP1, FADD, FAS, FASLG, FCGR1A, FCGR2A, FCGR2B, FCGR3A, FCGR3B, FCN3, FERMT3, FMR1, FOXD3, FOXN1, FOXP3, FPR1, G6PC3, GATA2, GFI1, HAX1, HLA-C, HLA-DQB1, HLA-DRA, HLA-DRB1, ICOS, IFIH1, IFNGR1, IFNGR2, IGLL1, IKBKB, IKBKG, IL10, IL10RA, IL10RB, IL12B, IL12RB1, IL17F, IL17RA, IL17RC, IL18, IL1B, IL1RN, IL21, IL21R, IL2RA, IL2RG, IL36RN, IL6, IL7R, INO80, IRAK4, IRF8, ISG15, ITCH, ITGAM, ITGB2, ITK, JAGN1, JAK3, KRAS, LAMTOR2, LIG4, LPIN2, LRBA, LYST, MAGT1, MALT1, MAP3K14, MASP1, MASP2, MCM4, MEFV, MOGS, MS4A1, MVK, MYD88, NBN, NCF1, NCF2, NCF4, NFAT5, NFKB1, NFKB2, NFKBIA, NHP2, NLRC4, NLRP1, NLRP12, NLRP2, NLRP3, NLRP7, NOD2, NOP10, NRAS, PARN, PAX4, PDCD1, PGM3, PIK3CA, PIK3CD, PIK3R1, PLCG2, PMS2, PNP, POLE, POMP, PRF1, PRKCD, PRKDC, PSMA3, PSMB4, PSMB8, PSMB9, PSTPIP1, PTPN22, PTPRC, PYCARD, RAB27A, RAC2, RAG1, RAG2, RBCK1, RFX5, RFXANK, RFXAP, RHOH, RNASEH2A, RNASEH2B, RNASEH2C, RNF168, RNF31, RORC, RPSA, RTEL1, SAMHD1, SBDS, SEMA3E, SERPING1, SH2D1A, SH3BP2, SIAE, SLC29A3, SLC35C1, SLC37A4, SMARCAL1, SP110, SPINK5, STAT1, STAT3, STAT5B, STIM1, STK4, STX11, STX1A, STXBP2, TAP1, TAP2, TAPBP, TAZ, TBK1, TCF3, TERT, TG, THBD, TICAM1, TINF2, TLR3, TLR4, TMC6, TMC8, TNFAIP3, TNFRSF11A, TNFRSF13B, TNFRSF13C, TNFRSF1A, TNFRSF4, TNFSF12, TPP2, TRAF3, TRAF3IP2, TREX1, TRNT1, TTC37, TTC7A, TYK2, UNC13D, UNC93B1, UNG, USB1, VPS13B, VPS45, WAS, WIPF1, XIAP, ZAP70, ZBTB24, ZFAT

Supplementary Figure S1. Next-generation (whole exome) sequencing and alignment visualization using Integrative Genomics Viewer (IGV); please refer to the main text for details.

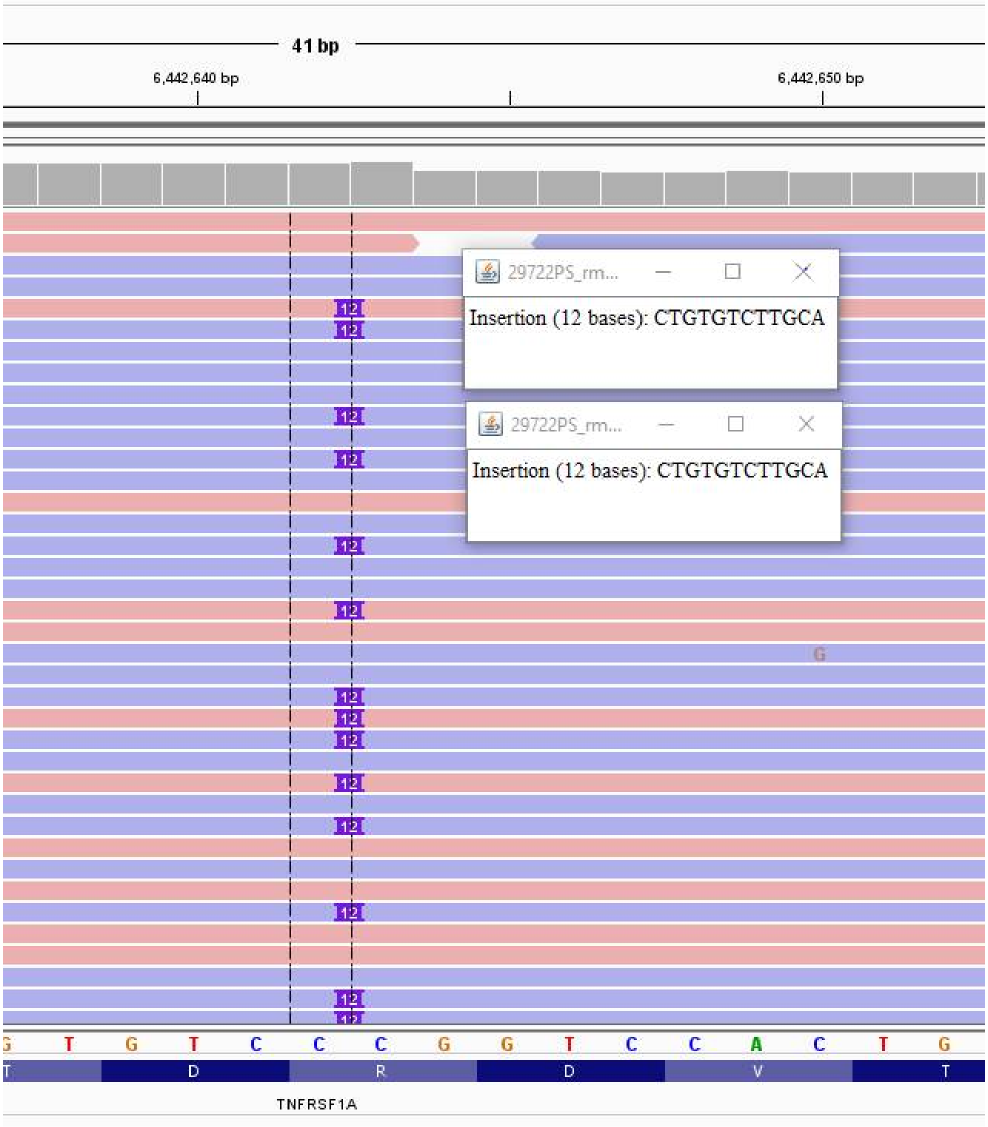

Supplement: Supplementary file 1 [file jcm-10-00465-s001.pdf]
